# Metabolomic assessment of mechanisms underlying anti-renal fibrosis properties of petroleum ether extract from *Amygdalus mongolica*

**DOI:** 10.1080/13880209.2021.1920619

**Published:** 2021-05-14

**Authors:** Chen Gao, Wan-fu Bai, Hong-bing Zhou, Hai-mei Hao, Ying-chun Bai, Quan-li Liu, Hong Chang, Song-li Shi

**Affiliations:** a Department of Pharmacy, Baotou Medical College, Inner Mongolia University of Science and Technology, Baotou, China; b Department of Pharmacy, The Second Affiliated Hospital of Baotou Medical College, Inner Mongolia University of Science and Technology, Baotou, China; c Institute of Bioactive Substance and Function of Mongolian Medicine and Chinese Materia Medica, Baotou Medical College, Inner Mongolia University of Science and Technology, Baotou, China

**Keywords:** Traditional Chinese medicine, renal fibrosis, biomarkers, metabolic pathway, mechanism research

## Abstract

**Context:**

The petroleum ether extract (PET) of *Amygdalus mongolica* (Maxim.) Ricker (Rosaceae) has an ameliorative effect on renal fibrosis (RF).

**Objective:**

To evaluate the antifibrotic effects of *A. mongolica* seeds PET on RF by serum metabolomics, biochemical and histopathological analyses.

**Materials and methods:**

Sixty male Sprague-Dawley rats were randomly divided into the sham-operated, RF model, benazepril hydrochloride-treated model (1.5 mg/kg) and PET-treated (1.75, 1.25, 0.75 g/kg) groups, and the respective drugs were administered intragastrically for 21 days. Biochemical indicators including BUN, Scr, HYP, SOD, and MDA were measured. Haematoxylin and eosin and Masson staining were used for histological examination. The serum metabolomic profiles were determined by UPLC-Q-TOF/MS and metabolism network analysis. Acute toxicity test was performed to validate biosafety.

**Results:**

The PET LD_50_ was >23.9 g/kg in rats. PET significantly alleviated fibrosis by reducing the levels of Scr (from 34.02 to 32.02), HYP (from 403.67 to 303.17) and MDA (from 1.84 to 1.73), and increasing that of SOD (from 256.42 to 271.85). Metabolomic profiling identified 10 potential biomarkers, of which three key markers were significantly associated with RF-related pathways including phenylalanine, tyrosine and tryptophan biosynthesis, amino sugar and nucleotide sugar metabolism and tyrosine metabolism. In addition, three key biomarkers were restored to baseline levels following PET treatment, with the medium dose showing optimal effect.

**Conclusions:**

These findings revealed the mechanism of *A. mongolica* PET antifibrotic effects for RF rats on metabolic activity and provided the experimental basis for the clinical application.

## Introduction

Renal fibrosis (RF) and chronic kidney disease (CKD) affect 50% of the adults over 70 years of age, and 10% of the global population (Boor et al. 2010; Schmitt 2012). RF is a set of various physiological and pathological changes that accompany CKD of different aetiologies and progress to end-stage renal failure (Chen et al. 2016). Therefore, delaying or reversing the fibrotic process in renal tissues is a promising therapeutic strategy against CKD (Nogueira et al. 2017). Currently, RF is mainly treated with angiotensin converting enzyme inhibitors (ACEI), angiotensin receptor antagonists, transforming growth factor β_1_ (TGF-β_1_) neutralising antibodies or natural antagonists. In addition, gene therapy approaches have also been tested in clinical trials. Traditional Chinese medicine (TCM) formulations have the advantages of pleiotropic action and minimal side effects, and are a promising alternative for the prevention and treatment of RF (Ellison 2017; Ji and He 2018). According to the TCM theory, the treatment of RF involves clearing and purging the turbidity. The mechanistic basis of TCM action is increasingly being elucidated through metabolomics and transcriptomics, and several promising biomarkers have been identified for the early diagnosis and treatment of fibrosis (Qi et al. 2014; Wu et al. 2017).

*Amygdalus mongolica* (Maxim.) Ricker (Rosaceae) of the flat peach family is a deciduous, drought-tolerant shrub endemic to the Mongolian plateau (Ma 1989; Zhao 1995). According to the Records of Inner Mongolia Plant Medicine, the seeds have medicinal properties and are used in the ‘Yu Li Ren’ (*Pruni semen*) TCM formulation (Si 2003) to relieve cough, resolve phlegm, moisten dry intestines and dry throat, and treat Yin deficiency constipation and edoema through its diuretic effects. In addition, *Pruni semen* is used as an auxiliary treatment for kidney disease and nephritis (Zhou & Zheng 1960). The chemical constituents of *A. mongolica* include unsaturated fatty acids, amygdalin, organic acids, proteins, flavonoids, polysaccharides, alkaloids, α-VE and other pharmaco-active compounds (Shi, Bai, et al. 2013; Shi, Niu, et al. 2013; Su et al. 2013; Zheng DH et al. 2013; Bai et al. 2015; Zheng et al. 2018). The petroleum ether extract (PET) of *A. mongolica* is rich in fatty acids such as oleic acid, palmitic acid, palmitic acid, linoleic acid, 8,11-octadecenedioic acid and arachidonic acid that can inhibit liver fibrosis by reversing lipid peroxidation (Zheng et al. 2016, 2018; Wu et al. 2017). Furthermore, *A. mongolica* extracts can also prevent pulmonary and renal fibrosis (Chang et al. 2020; Hao et al. 2020; Jia et al. 2020), and the reno-protective effects have been attributed to PET and *n*-butanol. However, the mechanisms underlying the antifibrotic effects of *A. mongolica* PET are still unclear. A previous study showed that the effective dose range of *A. mongolica* PET for hypolipidemic and antioxidant action is 0.5426–1.5 g/kg (Zheng et al. 2016, 2018). Furthermore, oral administration of 21.5 g/kg *A. mongolica* oil for 14 days did not induce any toxic reactions in a rat model (Zheng et al. 2017). A recent study showed that linoleic acid constitutes 67.56% of *A. mongolica* PET (Zheng et al. 2018). Since polyunsaturated fatty acids can effectively combat renal fibrosis in obstructive nephropathy by improving dyslipidemia, reducing lipid peroxidation and inflammation and inhibiting myofibroblast proliferation (Zhao et al. 2013; Han 2014), it is rational to surmise that linoleic acid is the major pharmaco-active component of *A. mongolica*. This study further investigates the therapeutic effects and potential mechanisms of *A. mongolica* PET in RF.

Metabolomics is the study of the metabolite profiles of cells, biological fluids and tissues, and their interactions. It is a high-throughput approach for screening novel biomarkers, and particularly useful for elucidating the multiple targets and metabolic pathways of TCM (Buriani et al. 2012; Zhou et al. 2014; Lyu et al. 2018). In this study, we established a rat model of RF by unilateral ureteral obstruction (UUO), and used metabolomics to explore the mechanism of PET action against RF. Our findings provide an experimental basis for the pharmacological analysis of other TCM formulations.

## Materials and methods

### Materials

The seeds of *A. mongolica* were collected from Alashanyabrai Gobi Inner Mongolia in 2018. The dry mature seeds were identified by Professor Songli Shi of Inner Mongolia University of Science and Technology Medical College of Baotou (Supplementary Figures S1 and S2). Benazepril hydrochloride was purchased from Beijing Novartis Pharmaceutical Co. Ltd., pentobarbital sodium from Merck (Germany) and sodium carboxymethyl cellulose from Tianjin Kaitong Chemical Reagent Co. Ltd. The penicillin sodium injection solution was from North China Pharmaceutical Co. Ltd. (batch number: F7116323). HE and Masson staining kits were purchased from Nanjing Jiancheng Technology Co. Ltd. LC-MS grade methanol (67–56–1) and acetonitrile (75–05–8) were obtained from Honeywell, and formic acid from SIGMA (64–18–6).

Grinder (MS-700, Ningbo Meishan Meixin Electrical Appliance Co., Ltd.); Rotary evaporation instrument (RE-52, Shanghai Yarong Biochemical Instrument Factory); Circulating water vacuum pump (SHZ-III, Shanghai Yarong Biochemical Instrument Factory); Biological tissue automatic dehydrator (NP-7, Xiaogan Nuopu Electronic Technology Co., Ltd.); Slicer (RM2235, Leica Germany); Biological tissue embedding machine (NP-B, Xiaogan Nuopu Electronic Technology Co., Ltd.); Microscope (CX31, Olympus Japan); Low-speed automatic balancing centrifuge (TDZ4-WS, Changsha Xiangyi Centrifuge Instrument Co., Ltd.); Automatic biochemical analyser (AU640, Olympus Japan); Fast mixer (K4896-B, Jiangyan Xinkang Medical Equipment Co., Ltd.); High-speed desktop freezing centrifuge (TGL-16M, Hunan Xiangyi Laboratory Instrument Development Co., Ltd.); Enzyme labelling instrument (Semel Fisher Technology (China) Co., Ltd.); Ultra high performance liquid chromatography (ExionLC, Sciex); High resolution mass spectrometry (TripleTOF 5600, Sciex); Pure water metre (Clear D24 UV, Merck Millipore); Chromatographic column (ACQUITY UPLC HSS T3 1.8 μm 2.1 × 100 mm, Waters).

### Preparation of petroleum ether extract from *Amygdalus mongolica*

The seeds of *A. mongolica* were peeled, deshelled and crushed, and extracted with 95% and 70% ethanol. The extraction conditions were as follows: temperature 70 °C, solid-to-liquid ratio 1:10, and time 2 h. The extracts were combined and concentrated under reduced pressure, and the total ethanol extract was uniformly dispersed in distilled water, extracted with petroleum ether, and concentrated under reduced pressure to obtain PET (28.5% yield). PET composition was analysed as previously described (Zheng et al. 2018), and the main fatty compounds were unsaturated fatty acids (73.2%) such as oleic acid (28.87%) and linoleic acid (38.69%), and saturated fatty acids like palmitic acid (19.66%).

### Acute toxicity test of A. mongolica PET

Ten male and female SD rats each (weighting 180–200 g) were acclimatised for 3–4 days and fasted overnight. The animals were given 23.9 g/kg PET once via the oral route and observed for 14 days for the clinical signs of toxicity (Zheng et al. 2017).

### Establishment of UUO model and treatment regimen

Sixty male SPF grade male Sprague-Dawley rats weighing 170–200 g were purchased from the Department of Medical Sciences of Peking University (Department of Experimental Animal Science; licence number SCXK (Beijing) 2011–0012). The animals were randomly divided into the sham-operated (SDG), model (MOD), benazepril-treated (BH), and the high (PET-H), medium (PET-M) and low (PET-L) dose PET groups (*n* = 10 each). RF was induced by unilateral ureteral ligation as described previously (Ma et al. 2018). The animals in the SDG and MOD groups were given daily oral gavage of 4 mL normal saline. The dosage of BH was 1.5 mg/kg/day, and that of PET-H, PET-M and PET-L were 1.75, 1.25 and 0.75 g/kg/day, respectively (Zheng et al. 2016, 2018). The drugs were administered for 21 days. The experimental protocol was approved by Medical Ethics Committee of Baotou Medical College of Inner Mongolia University of Science and Technology.

### Specimen collection

Twenty-four hours after the last drug administration, the animals were weighed and anaesthetised by intraperitoneal injection of 3% pentobarbital. Blood was drawn from the abdominal aorta and centrifuged at 3000 rpm for 10 min. The serum was collected for biochemical and metabolomic analyses. The left kidney lobe was cut into 1 cm^3^ pieces and fixed in 10% paraformaldehyde solution for HE and Masson staining, and the right lobe was frozen at −80 °C.

### Biochemical and histological examination

Serum levels of serum creatinine (Scr), blood urea nitrogen (BUN) and serum albumin (ALB) were measured by an automated analyser. The content of superoxide dismutase (SOD), malondialdehyde (MDA) and hydroxyproline (HYP) in the renal tissues were determined using specific kits as per the manufacturer’s instructions. For histological examination, the paraffin-embedded tissues were cut into 3–4 μm-thick sections and stained with HE and Masson dyes as per standard protocols. The sections were observed under a light microscope for pathological changes, and fibrotic injury was scored as previously described (Zhang, Li, et al. 2018).

### UPLC-Q-TOF/MS

The liquid phase gradient was as follows: 0–0.5 min − 5% B (acetonitrile, 0.1% formic acid) 95% A (water, 0.1% formic acid), 0.5–7 min − 5%–100% B, 7–8 min − 100% B, 8–8.1 min − 100% B, 8.1–10 min − 5% B; 0–0.5 min − 95% B, 0.5–9.5 min − 95% to 65% B, 9.5–10.5 min − 65%–40% B, 10.5–12 min − 40% B, 12–12.2 min − 40%–95% B, 12.2–15 min − 95% B. The injection volume for each sample was 4 µL. The column temperature was maintained at 35 °C and the flow rate was 0.4 mL/min.

The mass spectrometry (MS) conditions were as follows: ion source Gas1 − 60 PSI, ion source Gas2 − 60 PSI, interface heating temperature −650 °C, ion spray voltage in positive ion mode −500 V, ion spray voltage in negative ion mode −4500 V. MS data were collected in the IDA mode. The TOF quality scan range was 60–1200 Da and was completed within 150 milliseconds with a total cycle time of 0.56 s. A 40 GHz four-anode/channel multi-channel TDC detector was used to monitor the scans, and the four times of each scan were added when the pulse frequency was 11 kHz.

### Data analysis

The raw MS data was converted to mzXML format using the MSConvert software. The XCMS program was then used for data processing, including peak selection, peak grouping, retention time correction, second peak grouping, and isotope and adduct extract annotation. The metabolites were annotated using online KEGG and HMDB databases by matching the exact molecular mass data (*m/z*) of samples with the standards. Finally, the identified metabolites were identified using an in-house fragment spectrum library. The multivariable data matrix was analysed by SIMCA-P 14.1 software (Umetrics AB, Umeå, Sweden). Principal component analysis (PCA) and partial least squares discriminant analysis (PLS-DA) were used to visually analyse the clustering results, and the potential biomarkers of RF were screened based on variable importance of projection (VIP) > 1 and *p* value (*t*-test) < 0.05. The metabolic pathways of differential metabolites were analysed by MetPA with MetaboAnalyst 4.0 software, and the key RF-related pathways were screened on the basis of impact > 0.02 and − log (*P*) > 2. The diagnostic power of the putative biomarkers was determined by the ROC test, the KEGG pathway database was used for network analysis. SPSS17.0 was used to analyse the experimental data that were expressed as $\bar{\text{x}}\pm \text{s}.$ The means of different groups were compared by single factor analysis of variance (*p* < 0.05).

## Results

### Acute toxicity test

No apparent symptoms were detected following oral administration 23.9 g/kg PET for 14 days, indicating that it is non-toxic according to the ‘Food Safety Toxicity Evaluation Procedures and Methods’ (Zheng et al. 2017).

### Determination of biochemical indicators and histopathological analysis

**Figure 1.**
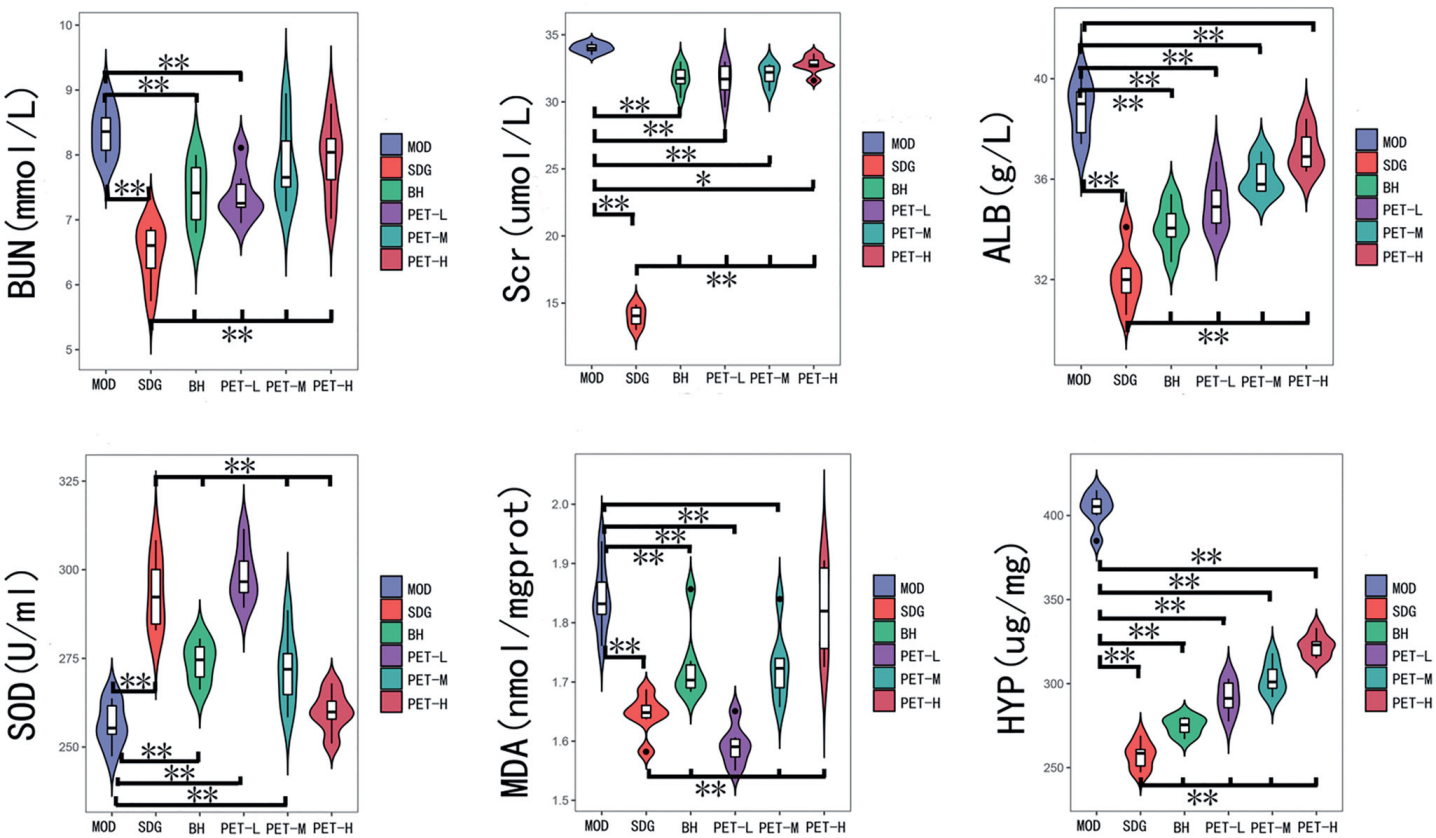
Effect of *A. mongolica* PET on the physiological and biochemical indices of the RF model. All values represent the mean ± SD. *p < 0.05, **p < 0.01.

The serum levels of BUN, Scr and ALB, and the content of MDA and HYP in the renal tissues were significantly higher in the MOD versus SDG groups, whereas the renal SOD activity was significantly reduced in the former (*p* < 0.01), indicating successful RF modelling. Treatment with BH and the low and medium doses of PET restored all indices to normal levels (*p* < 0.01; Figure 1; Table 1), thereby showing reno-protective and antioxidant effects.

**Table 1.** Changes in content of *A. mongolica* PET on the physiological and biochemical indices of the RF model.

	BUN	Scr	ALB	SOD	MDA	HYP
MOD	8.36 ± 0.39^##^	34.02 ± 0.36^##^	38.77 ± 1.10^##^	256.42 ± 6.30^##^	1.84 ± 0.06^##^	403.67 ± 10.50^##^
SDG	6.48 ± 0.45**	14.02 ± 0.77**	32.10 ± 1.19**	293.45 ± 10.35**	1.64 ± 0.04**	257.17 ± 8.16**
BH	7.41 ± 0.50**^##^	31.77 ± 0.95**^##^	34.10 ± 0.94**^##^	273.94 ± 5.77**^##^	1.73 ± 0.07**^##^	274.67 ± 5.35**^##^
PET-L	7.40 ± 0.41**^##^	31.60 ± 1.31**^##^	35.02 ± 1.08**^##^	298.50 ± 8.05**	1.59 ± 0.03**	291.83 ± 10.07**^##^
PET-M	7.88 ± 0.67^##^	32.02 ± 0.80**^##^	36.08 ± 0.70**^##^	271.85 ± 10.73**^##^	1.73 ± 0.06**^##^	303.17 ± 9.56**^##^
PET-H	7.95 ± 0.62^##^	32.75 ± 0.67*^##^	37.13 ± 0.84**^##^	259.97 ± 5.77^##^	1.82 ± 0.08^##^	322.33 ± 6.80**^##^

All values represent the mean ± SD. *p < 0.05 and **p < 0.01 compared to the model group. ^#^*p* < 0.05 and ^##^*p* < 0.01 compared to the sham group.

**Figure 2.**
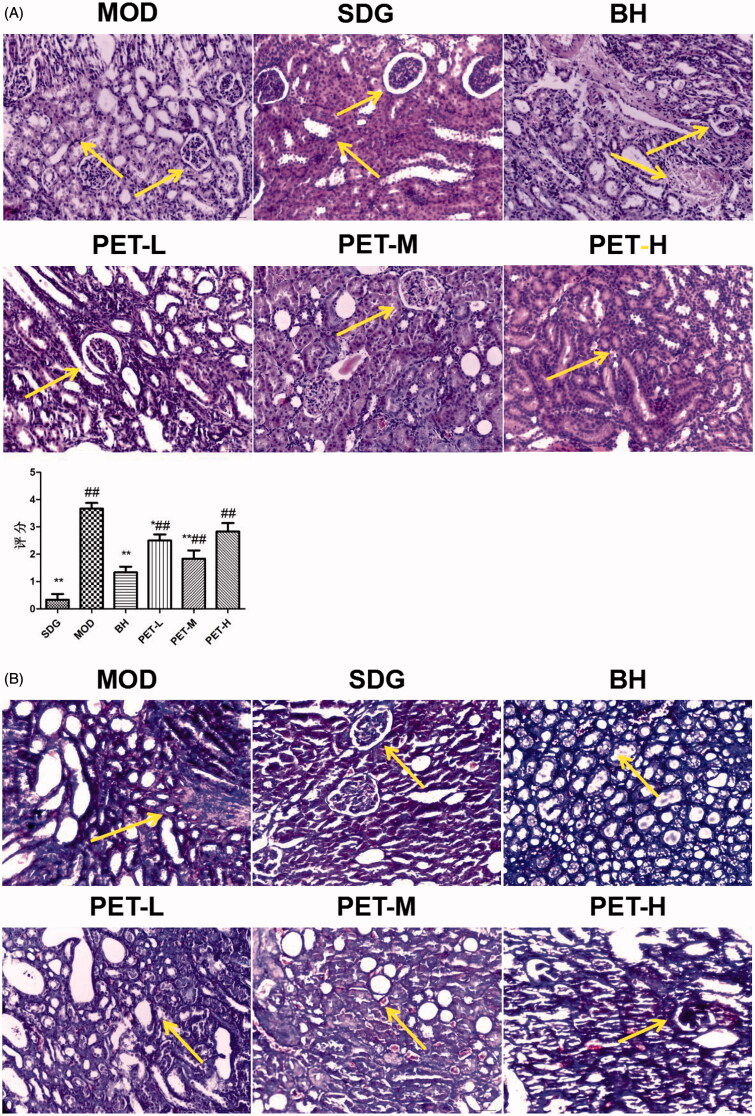
Pathological changes in renal tissue: (A) Representative images of HE staining (×200) and score; (B) Representative images of Masson staining (×200). Values represent the mean ± SD. *p < 0.05 and **p < 0.01 compared to the model group. ^#^*p* < 0.05 and ^##^*p* < 0.01 compared to the sham group.

As shown in the H&E and Masson-stained tissue images in Figure 2, there were no significant histopathological changes in the tubule-interstitium of the kidneys in the SDG rats. In contrast, UUO modelling resulted in markedly reduced glomeruli, significant atrophy of renal tubules with bare basement and interstitial widening, massive infiltration of inflammatory cells, and focal fibrosis. Treatment with BH, PET-L, PET-M and PET-H reduced interstitial inflammation and fibrosis, and mitigated the damage to some renal tubules as well. PET-M showed the most significant effect (*p* < 0.01). Taken together, both PET and BH can protect kidney tissues from UUO-induced fibrosis.

### Multivariate data analysis

**Figure 3.**
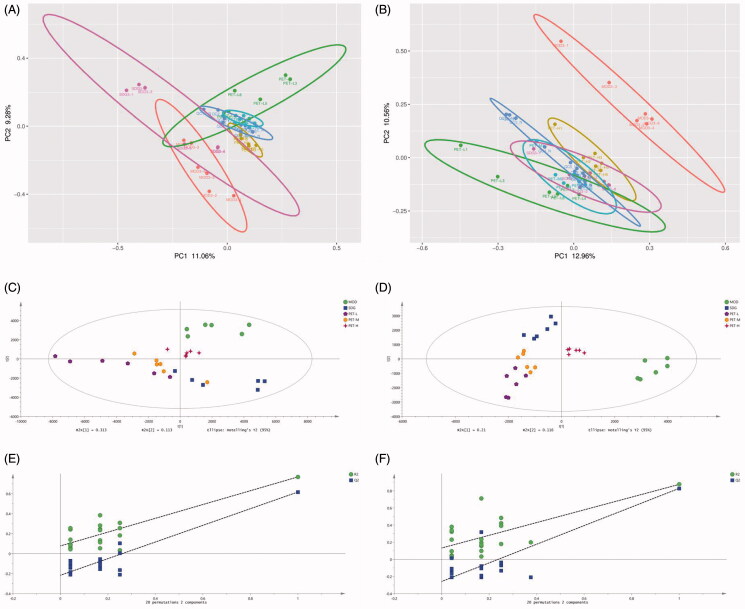
PCA score plot, PLS-DA score plot and validation plot of the PLS-DA model of SDG, MOD, BH, PET-L, PET-M and PET-H. (A) PCA score plot in positive ion mode; (B) PCA score plot in negative ion mode; (C) PLS-DA score plot in positive ion mode; (D) PLS-DA score plot in negative ion mode; (E) Validation plot of the PLS-DA model in positive ion mode; (F) Validation plot of the PLS-DA model in negative ion mode.

Serum samples of each group were assessed by principal component analysis (PCA) and partial least squares discrimination analysis (PLS-DA) in positive and negative ion mode (Figure 3). As shown in the PCA score plots (Figure 3(A,B)), the samples of each group were well clustered in the unsupervised mode. In addition, the MOD and SDG showed greater distinction in the anion mode, while PET-L, PET-M and PET-H were distinct from MOD and closer to SDG. PLS-DA on the other hand showed further separation of MOD from SDG, PET-L, PET-M and PET-H, while the treatment groups were closer to SDG and PET-M was most similar to SDG (Figure 3(C,D)). The replacement test diagram was used to assess PLS-DA overfitting (Figure 3(E,F)), which showed that R^2^ and Q^2^ from left to right was lower than the original value on the far right and Q^2^ intersected with Y axis in the negative half axis, indicating good fitting and reliable results.

### Screening and identification of potential biomarkers

**Figure 4.**
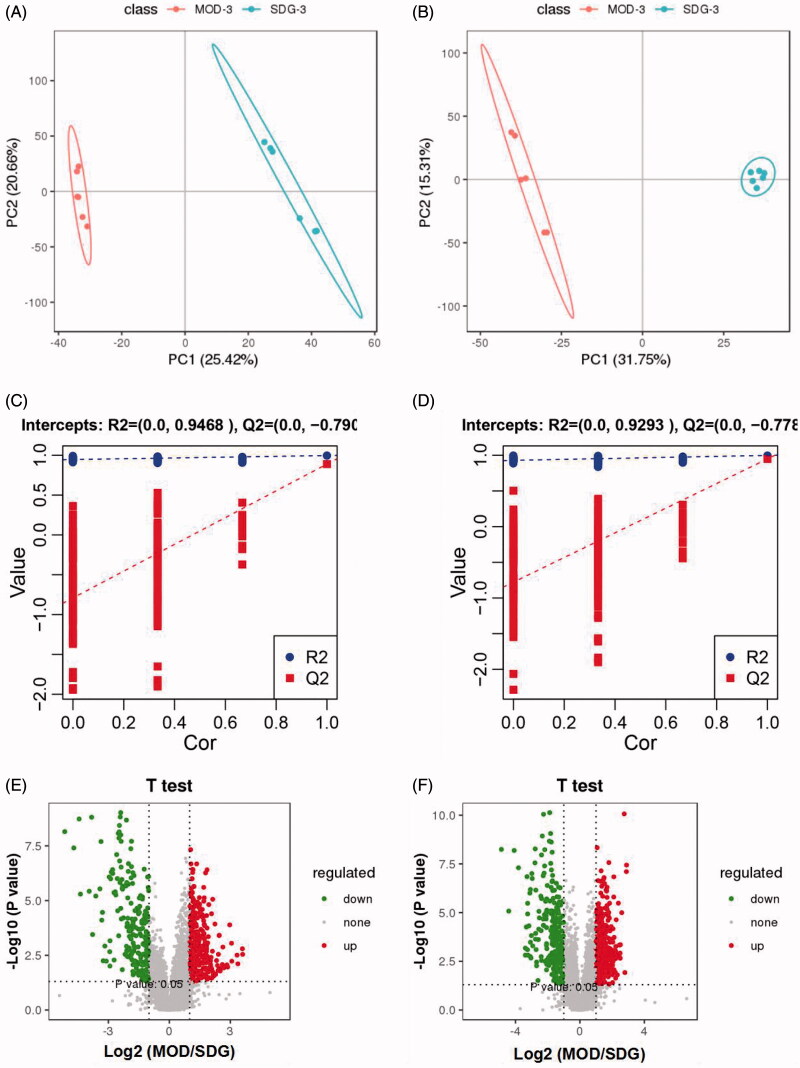
PLS-DA score plot, validation plot of the PLS-DA model and Volcano Plot map of SDG and MOD. (A) PLS-DA diagram in positive ion mode; (B) PLS-DA diagram in negative ion mode; (C) validation plot of the PLS-DA model in positive ion mode; (D) validation plot of the PLS-DA model in negative ion mode; (E) Volcano Plot map in positive ion mode; (F) Volcano Plot map in negative ion mode.

**Figure 5.**
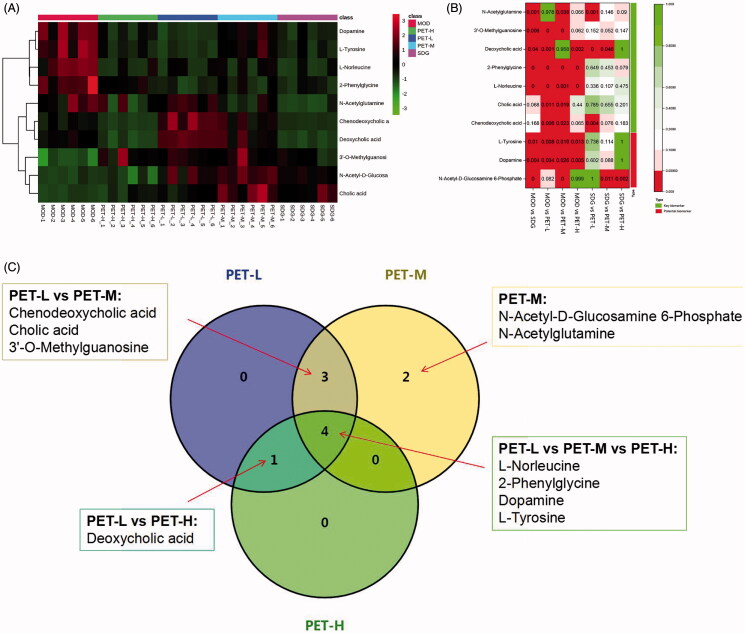
Analysis of the identified potential biomarkers. (A) Heat map of the differentially abundant metabolites in all groups. Rows – samples; columns – metabolites. The degree of colour saturation indicates the metabolite expression with green and red respectively indicating lowest and highest expression. (B) *p*-Value heat map of the differential abundance of metabolites in all groups. Rows – samples; columns – metabolites. The degree of colour saturation indicates intergroup differences in metabolite expression values with green and red respectively indicating non-significant and significant difference. (C) VENN diagram of PET-L, PET-M and PET-H biomarkers.

As shown in the PLS-DA score plots (Figure 4(A,B)), MOD and SDG had good differentiation in positive and negative ion modes, which indicated distinct metabolomic profiles. In addition, the class permutation tests (Figure 4(C,D)) indicated good fitness and predictive values of these models. Ten differentially abundant metabolites (eight in positive ion mode and two in negative ion mode) were identified in the model group relative to the control based on VIP > 1 and *p* < 0.05 (Table 2). As shown in the heat maps in Figure 5(A), the metabolites that increased (red) or decreased (green) in the model group were restored to normal levels by the different doses of PET.

**Table 2.** Identification of potential biomarkers.

No.	*m/z*	RT	ratio	t.test_ *p*.value	VIP	RankMS2hmdb	Common name	Chemical formula	Regulated
Positive ion mode									
1	391.28	170.55	0.24	1.98E-03	3.02	HMDB00619	Cholic acid	C24H40O5	Down
2	132.10	68.513	3.16	3.88E-04	2.75	HMDB01645	L-Norleucine	C6H13NO2	Up
3	151.06	86.41	2.30	1.22E-04	2.55	HMDB02210	2-Phenylglycine	C8H9NO2	Up
4	357.28	186.35	2.65	1.59E-05	2.40	HMDB00518	Deoxycholic acid	C24H40O4	Up
5	315.13	166.82	0.43	1.90E-02	2.38	HMDB06038	3′-*O*-Methylguanosine	C11H15N5O5	Down
7	136.07	66.23	2.04	6.51E-05	2.19	HMDB00073	Dopamine	C8H11NO2	Up
8	182.08	66.23	2.03	1.04E-04	2.19	HMDB00158	L-Tyrosine	C9H11NO3	Up
10	153.06	90.69	2.66	1.09E-03	1.97	HMDB06029	*N*-Acetylglutamine	C7H12N2O4	Up
Negative ion mode									
1	783.56	186.87	4.12	8.64E-04	2.13	HMDB00518	Chenodeoxycholic acid	C24H40O4	Up
2	317.07	129.86	0.38	9.41E-03	2.11	HMDB01062	*N*-Acetyl-D-Glucosamine 6-Phosphate	C8H16NO9P	Down

The heat map of the changes in metabolite contents showed that PET-L normalised chenodeoxycholic acid, cholicacid, l-norleucine, 2-phenylglycine, deoxycholic acid, 3′-*O*-methylguanosine, dopamine and l-tyrosine, whereas PET-M restored chenodeoxycholic acid, *N*-acetyl-d-glucosamine 6-phosphate, cholic acid, l-norleucine, 2-phenylglycine, 3′-*O*-methylguanosine, dopamine, l-tyrosine and *N*-acetylglutamine. Interestingly, PET-H only restored 5 metabolites, including l-norleucine, 2-phenylglycine, deoxycholic acid, dopamine and l-tyrosine (*p* < 0.05; Figure 5(B)).

Moreover, we compared the concentrations of potential biomarkers among all groups and demonstrated that biomarkers were normalised or reversed by the treatment with PET, respectively. Venn-diagram (Figure 5(C)) shows the results of number of potential biomarkers for significant callbacks in each group, PET-H, PET-M and PET-L could respectively callback 5, 9, and 8 potential biomarkers, and simultaneously act on 4 same potential biomarkers together. Taken together, RF significantly alters the abundance of several metabolites, which are potential diagnostic biomarkers.

### Diagnostic potential and biological function of RF-related metabolites

**Figure 6.**
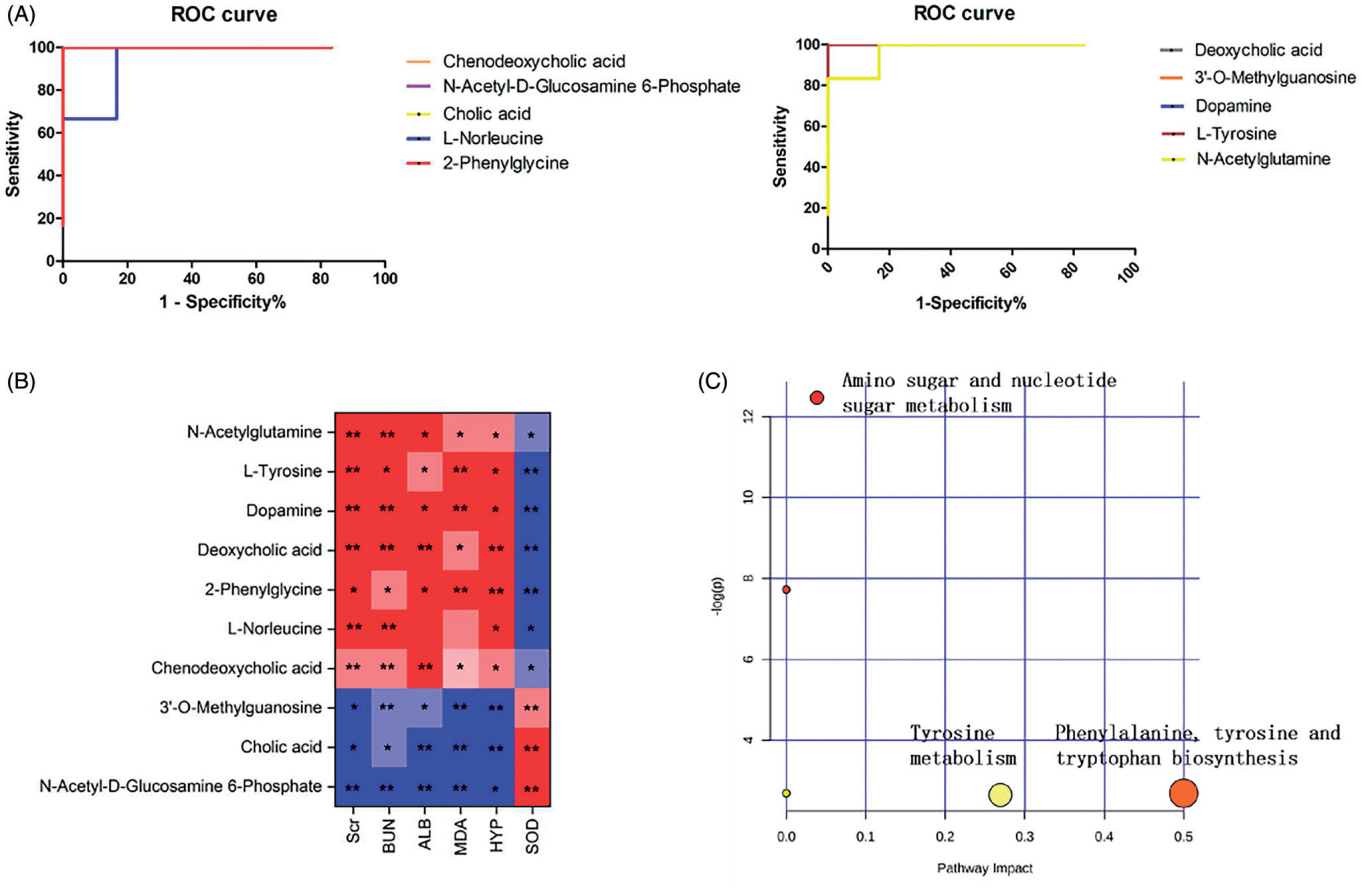
Analysis of related metabolic pathways and crucial biomarkers. (A) ROC analysis of 10 potential biomarkers; (B) Pearson rank correlation analysis between 10 crucial biomarkers and biochemical indicators. The red and blue colour gradients represent positive and negative correlation respectively. All values represent the mean ± SD. *p < 0.05, **p < 0.01; (C) Metabolic pathway analysis of crucial biomarkers. Summary of the altered metabolism pathways determined with MetPA and MetaboAnalyst 4.0. The size and colour of each circle indcate the pathway impact value and *p*-value respectively.

ROC analysis was performed on the 10 potential biomarkers to determine their diagnostic potential. As shown in Figure 6(A), the AUC values of all biomarkers were greater than 0.9, indicating high diagnostic accuracy.

Subsequently, a Pearson rank correlation analysis was performed to directly measure the correlations between these biomarkers and biochemical indicators. As shown in Figure 6(B), serum Scr, BUN, ALB and MDA, and renal HYP were positive correlated with l-norleucine, 2-phenylglycine, l-tyrosine, dopamine, deoxycholic acid, chenodeoxycholic acid and *N*-acetylglutamine, and negatively with *N*-acetyl-d-glucosamine 6-phosphate, cholic acid and 3′-*O*-methylguanosin. SOD was positively correlated with l-norleucine, 2-phenylglycine, l-tyrosine, dopamine, deoxycholic acid, chenodeoxycholic acid, and *N*-acetylglutamine, and negatively with *N*-acetyl-d-glucosamine 6-phosphate, cholic acid, 3′-*O*-methylguanosine.

MetPA was next performed to identify the crucial biomarkers among the ten potential metabolites and the metabolic pathways most affected by RF. As shown in Figure 6(C), three metabolic pathways involved in amino sugar and nucleotide sugar metabolism, phenylalanine, tyrosine and tryptophan biosynthesis and tyrosine metabolism were markedly dysregulated (-Log(*P*) >2 and impact >0.02). These pathways are considered are closely associated with the occurrence and development of RF. Finally, three key serum metabolites were identified from these pathways.

## Discussion

Renal fibrosis is a complex pathological process, and metabolomic techniques can help screen for sensitive biomarkers and analyse endogenous metabolites *in vivo* to elucidate its underlying mechanisms. We established a rat model of RF by UUO, and used pharmacodynamics and metabolomics to study the antifibrotic mechanisms of *A. mongolica* PET. Benazepril, an angiotensin II inhibitor that reduces plasma aldosterone levels, increases vasodilatation, lowers blood pressure, relieves oxidative stress, increases glomerular filtration rate and reduces proteinuria (Wang et al. 2006; Zhang et al. 2013), was used as the positive control. The reno-protective effects of the different drugs were analysed on the basis of biochemical indices. Serum BUN and Scr levels are indicators of the extent of renal damage and fibrosis (Orchard et al. 2001; Lijnen 2005). The pathological basis of renal fibrosis are oxidative stress and inflammation (He et al. 2018). MDA, the final product of free radical-induced lipid peroxidation, is elevated in fibrotic lesions (Li et al. 2012). In addition, the content of HYP is closely related to the severity of renal interstitial fibrosis (Xiao et al. 2015). We found that different doses of *A. mongolica* PET reduced the levels of serum BUN, Scr and ALB, and the *in situ* content of MDA and HYP, and increased SOD activity. Thus, PET can delay the fibrotic process by alleviating oxidative damage, which was also corroborated by the significant improvement in the histopathological indices of fibrosis (Xue et al. 2004). A previous study showed that the effective dose range of *A. mongolica* PET for its hypolipidemic and antioxidant action is 0.5426–1.5 g/kg. Since the high dose (1.75 g/kg) used in our study was higher than the maximum effective dose, the medium dose (1.25 g/kg) was more effective in delaying and mitigating renal fibrosis.

**Figure 7.**
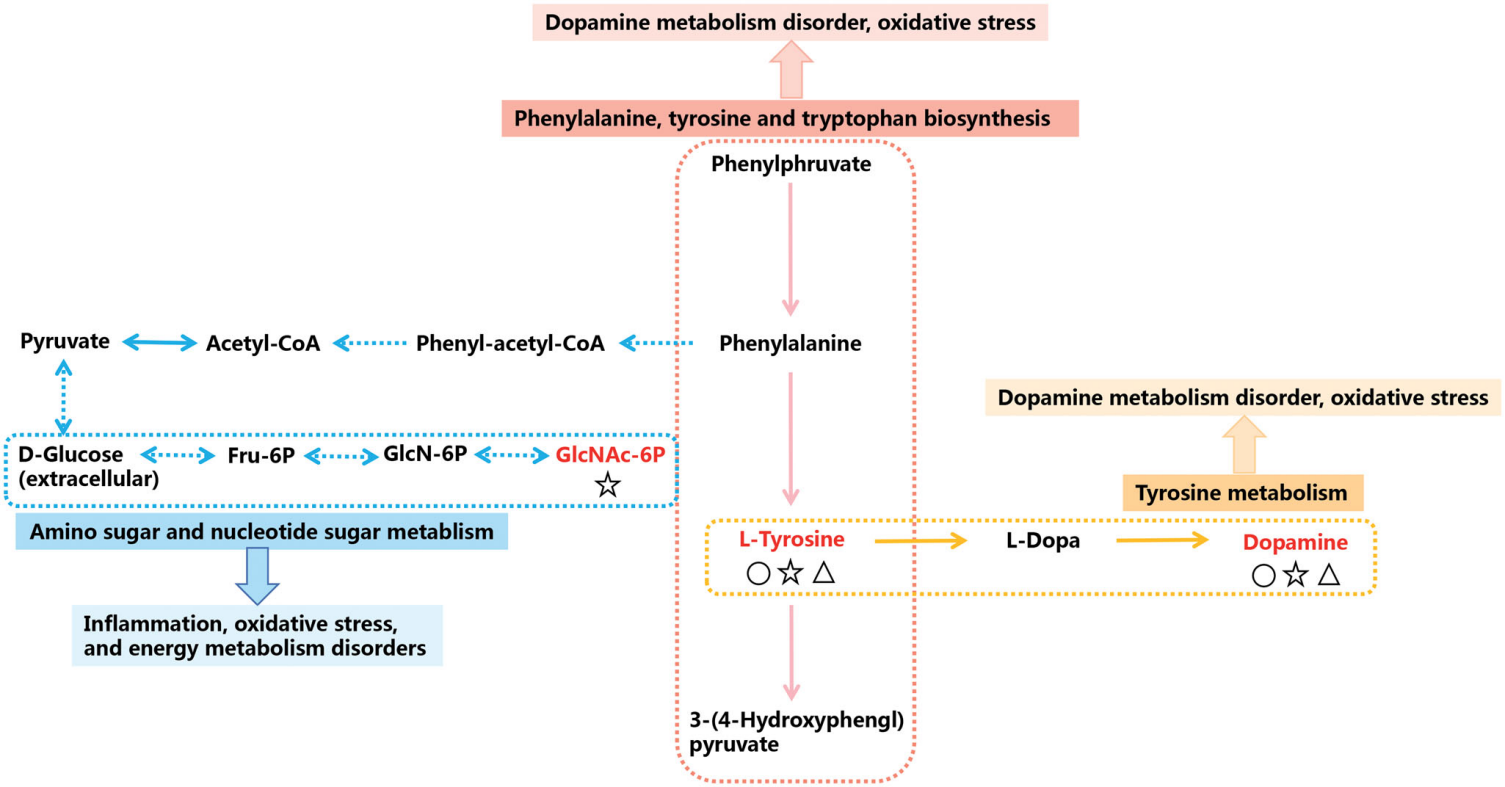
Network of the identified key biomarkers and pathways according to the KEGG pathway database. The metabolites coloured red represent the key biomarkers affecting the occurrence and development of renal fibrosis. The circles represent key biomarkers for PET-L callbacks. Pentacles represent a key biomarker of PET-M call-back. Triangles represent key biomarkers for PET-L callbacks.

Metabolomics is a powerful tool for evaluating drug efficacy and mechanism of action. Metabolomics analyses showed that PET mainly targets amino sugar and nucleotide sugar metabolism, phenylalanine, tyrosine and tryptophan biosynthesis and tyrosine metabolism, which are affected during renal fibrosis and lead to aberrant levels of l-tyrosine, dopamine and *N*-acetyl-d-glucosamine 6-phosphate (Figure 7). The medium PET dose was effective on all three metabolic pathways and restored the key biomarkers, and was therefore the optimal dose for antifibrotic effects.

l-Tyrosine is a non-essential amino acid and a precursor of various bioactive molecules, including brain catecholamine neurotransmitters including norepinephrine and dopamine (DA) (Wang et al. 2019). DA is an endogenous nitrogen-containing organic compound that is the intermediate product of l-tyrosine biosynthesis from dihydroxyphenylalanine. Gülçin (2007) showed that l-tyrosine scavenged free radicals like DPPH (1,1-diphenyl-2-picrylhydrazyl radical), ABTS, superoxide anion and H2O2, and also reduced ferrous ion chelation. Øvrehus et al. (2019) found that 11 amino acids, including tyrosine, phenylalanine, dopamine, homocysteine and serine, were affected in hypertensive nephrosclerosis patients. This disorder is characterised by a 30–70% decrease in urine output, endothelial dysfunction, atherosclerosis and renal fibrosis due to dysregulated dopamine biosynthesis in the kidney (Zhang 2013).

*N*-Acetyl-d-glucosamine 6-phosphate (GLCNAC6P) is involved in the metabolism of amino sugars and nucleotides. It is produced following glycolysis of *N*-acetyl-d-glucosamine by *N*-acetyl-d-glucosaminidase (NAG). NAG is abundant in the lysosomes of renal proximal tubule cells, and a marker of albuminuria and microalbuminuria in patients with type 2 diabetes mellitus, as well as of renal injury, inflammation and oxidative stress (Chang et al. 2020). Taken together, *A. mongolica* PET alleviates oxidative stress and inflammation in the kidney, and delays renal fibrosis by restoring amino sugar and nucleotide sugar metabolism.

## Conclusions

*Amygdalus mongolica* PET inhibited RF and improved renal function by restoring three key biomarkers and three metabolic pathways, which are likely involved in inhibiting release of inflammatory factors, reducing oxidative stress response, and regulating energy metabolism disorder. This study provides the experimental basis for the clinical application of *A. mongolica* PET.
